# Endometriosis Knowledgebase: a gene-based resource on endometriosis

**DOI:** 10.1093/database/baz062

**Published:** 2019-06-05

**Authors:** Shaini Joseph, Smita D Mahale

**Affiliations:** 1 ICMR-Biomedical Informatics Center, National Institute for Research in Reproductive Health, J.M. Street, Parel, Mumbai, India; 2 Division of Structural Biology, National Institute for Research in Reproductive Health, J.M. Street, Parel, Mumbai, India

## Abstract

Endometriosis is a complex, benign, estrogen-dependent gynecological disorder with an incidence of ~10% women in reproductive age. The implantation and growth of endometrial cells outside the uterus leads to the development of endometriosis. Endometriosis is also associated with comorbid conditions like cardiovascular and autoimmune diseases. The absence of non-invasive diagnostic markers, delayed diagnosis, high risk of recurrence of the disease on surgical removal of the tissue and absence of a definitive cure for endometriosis makes it imperative to gain insights into the complex etiology of endometriosis. A plethora of genes identified from blood and endometrial biopsies, involved in different pathways like steroid metabolism, angiogenesis, inflammation, etc. have been associated with endometriosis. However, the exact mechanism and genetic etiology of endometriosis still remain unclear. The polygenic nature of the disease, incongruent phenotypic manifestations in different ethnic populations and information scattered in literature makes it difficult to delineate the sub-network of genes that will aid in disease diagnosis and effective treatment. Endometriosis Knowledgebase is a manually curated database with information on genes associated with endometriosis. It holds information on 831 genes, their associated polymorphisms, gene ontologys, pathways and diseases. Genes in the database are enriched in pathways important for cell signaling, immune regulation and reproduction. A genetic overlap is seen between endometriosis and cancers, endocrine/reproductive, nervous system, immune and metabolic diseases. Network analysis of genes in the Endometriosis Knowledgebase helped predict 13 new candidate genes for endometriosis. These genes were found to be enriched in biological processes associated with endometriosis. The Endometriosis Knowledgebase and incorporated tools for gene and sequence-based analysis will benefit both researchers and clinicians working in the realm of reproductive biology.

## Introduction

Endometriosis is a common, benign, estrogen-dependent, gynecological disorder affecting ~10% of reproductive age women across the globe (1). It is associated with chronic pelvic pain, subfertility, dysmenorrhea, dyspareunia (2) leading to substantial socioeconomic burden and affecting the quality of life (3). A delay of 8–10 years in the diagnosis of endometriosis expected due paucity of reliable non-invasive diagnostic methods (4, 5). An increased risk of developing comorbid conditions like diabetes mellitus, cardiovascular disease, chronic liver disease, pelvic inflammatory diseases and autoimmune disorders such as rheumatoid arthritis (RA), systemic lupus erythematosus (SLE), Sjogren’s syndrome (6), multiple sclerosis (7), inflammatory bowel diseases (such as Crohn’s disease and ulcerative colitis) (8) and celiac disease (9) is observed in women with endometriosis.

The etiology of endometriosis is multifactorial in origin and is often referred to as ‘disease of theories’. Sampson’s theory of retrograde menstruation (RM) is the most commonly accepted theory, which proposes the reflux of viable endometrial cells into the peritoneal cavity leading to the development of endometriotic lesions (10). However, RM is observed in 70–90% of women and endometriosis occurs in only 7–10% of women suggesting the role of other factors in the etiology of endometriosis (11).

Several studies have found a familial aggregation of endometriosis (12–14). The largest twin study among 3096 Australian female twins estimated heritability of ~50% (15). These reports reinforce the role of genes in the pathogenesis of endometriosis.

Both candidate gene-based and high-throughput approaches like Genome-Wide Association Studies (GWAS) (16) have been employed to delineate genes associated with endometriosis. Genes mainly involved in inflammatory processes (e.g. *IL1, CCRL2, CCL17*) (17–19), steroid-synthesis (e.g. *HSD17B7, CYP19A1, HSD17B1*) (20, 21), detoxification (e.g. *GSTM1, GSTP1, GSTT1*) (22), hormone receptors (e.g. *PR, AR*) (23), estrogen metabolism (e.g. *ESR1*) (24), growth factors (e.g. *GDF15*) (25), adhesion molecules (e.g. *ICAM*) (26), apoptosis (e.g. *NFKB1, TNFRSF1A*) (27, 28), cell-cycle regulation (e.g. *CCNB1*) (29) and oncogenes (e.g. *EGFR, FOS*) (30, 31) were found to be associated with endometriosis. However, these studies were unsuccessful in providing replicable results when evaluated in larger independent cohorts (32).

The absence of a permanent cure for endometriosis, side effects of existing drugs that help to reduce the symptoms of the disease and a reported 35–50% chance of recurrence post-surgery makes developing diagnostic markers and identifying therapeutic targets for endometriosis a necessity (33–36).

The genetic complexity of endometriosis and its associated comorbid conditions suggest the need for use of an integrated approach to understand the pathophysiology of the disease and delineate its genetic etiology. A knowledgebase with manually curated information on endometriosis-associated genes, their ontologys, pathways and comorbid conditions will help to accelerate the research focused on understanding the etiology and pathogenesis of the disease. The knowledgebase would also be an excellent resource for the design of future research programs on endometriosis. The Endometriosis Knowledgebase is a comprehensive resource developed with an aim to augment research on endometriosis.

### Data collection

The aim of Endometriosis Knowledgebase was to develop a resource with all genes associated with endometriosis reported in the literature. The PubMed (37) database was queried for ‘Endometriosis’. The search was limited to human studies only. The genes (37) and single nucleotide polymorphisms (SNPs) (37) linked to the articles were retrieved from the Gene and SNP databases at National Center for Biotechnology Information (NCBI). A search query was built by combining each gene with its alternate names and the ‘endometriosis’ keyword. The query was further used to search the PubMed database. The articles retrieved were manually curated for information on the genes associated with endometriosis. The information mined from the PubMed reference is similar to information present in the CBD database (38). Each relevant PubMed reference abstract was mined for information on study population (population size, ethnicity), polymorphisms/mutations reported and other genes in the reference studied for their role in endometriosis. Genes having a role in endometriosis-associated infertility were annotated accordingly.

SNPs associated with endometriosis were retrieved from the SNP database of NCBI. Protein-related information was obtained from the UniProt database (39) and Gene Ontology (GO) information from the AMIGO2 database was mined using the GOOSE tool (40–43). The pathway information on the genes was retrieved from the KEGG Pathway database (44). The genes were also searched for disease associations in the Genetic Association Database (GAD) (45), KEGG disease database and in the Online Mendelian Inheritance in Man (OMIM) database (46). The information retrieved from the PubMed, Gene, SNP, KEGG, GAD, OMIM, UniProt and GO were compiled together as the Endometriosis Knowledgebase.

### Database architecture

Endometriosis Knowledgebase is built on Apache HTTP Server 2.2.11. The database is created using MySQL Server 5.1.33 and the web interfaces are designed using PHP 5.2.9, HTML and JavaScript. These are platform-independent, open source software packages.

### Database design

The knowledgebase consists of information on 831 genes and their 302 SNPs, 7032 gene ontologys, 367 pathways and 1390 diseases. The database homepage gives a brief introduction to the database. The user-friendly search and browse options allow efficient data retrieval. Gene and sequence-based tools have been incorporated in the knowledgebase for the benefit of users. These tools can be accessed through the Tools, BLAST and the Analysis link in the database interface. These options are described below:

**Tools:** This section allows users to identify conserved domains and motifs in their gene of interest using the CDART (47) or Motif Scan (48) link, respectively. The orthologs and SNPs link under the tools section help in retrieving known orthologs and variant effects of SNPs respectively using the g:Profiler program (49). The STRING (50) link allows users to see the known and predicted interactions between the user-selected set of genes.

**Blast** (37, 51)**:** Users can find homologous protein or nucleotide sequences for the user-selected set of genes using the BLAST link.

**Analysis:** The analysis tab in the knowledgebase allows users to cluster genes based on diseases, pathways and gene ontologys present in Endometriosis Knowledgebase separately. It also allows parsing user-selected genes to the Panther and g:profiler interface using the Panther (42) and Function (49) links, respectively.

**Figure 1:**
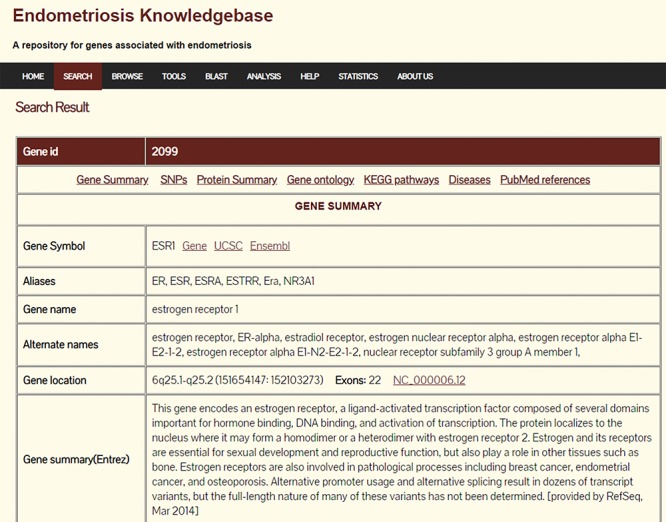
Search results page of Endometriosis Knowledgebase.

**Figure 2:**
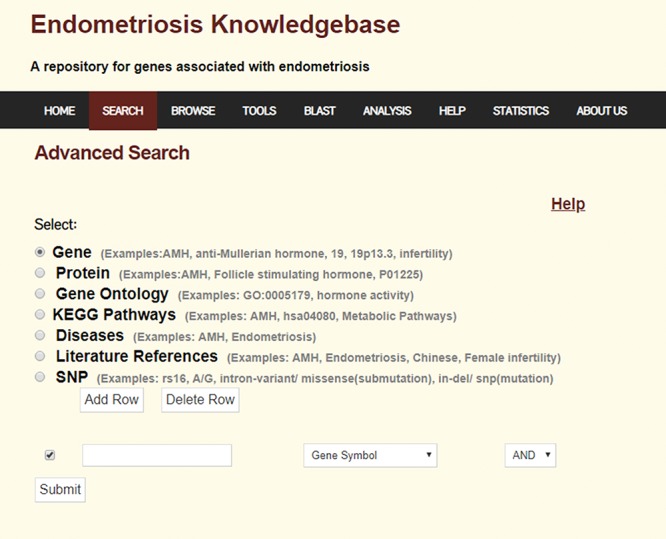
Advanced search page of Endometriosis Knowledgebase.

The help page guides the users in the usage of the database. The database statistics can be accessed through the Statistics link. A snapshot of the database results page and the advanced search page are shown in Figures 1 and 2, respectively.

### Data analysis

The genes present in the knowledgebase were analyzed to identify processes and pathways enriched in endometriosis using the Panther Gene List Analysis tool. The knowledgebase was found to be enriched in cell-signaling molecules and transcription factors indicating a regulatory role for most of the genes. Gonadotropin-releasing hormone receptor pathway, inflammation mediated by chemokine and cytokine signaling, the CCKR signaling pathway, angiogenesis and the integrin signaling pathway were found to be the most represented pathway among genes associated with endometriosis (42). Synchronized regulation of these pathways are important for cell survival, normal secretion of gonadotropins LH and FSH, pubertal development, reproduction, angiogenesis, trafficking and migration of immune cells etc. (42). The dysregulation of the gonadotropin-releasing hormone receptor pathway may result in high secretion of estrogen, a characteristic of patients with endometriosis. Aberrations in genes in the angiogenesis pathways lead to an imbalance in the expression of pro-angiogenic and anti-angiogenic factors that facilitate implantation, growth and/or survival of the endometriotic tissue in patients suffering from endometriosis (52). The dysregulation of the integrin signaling pathway expedites the establishment of endometriotic focii in endometriosis (53). These observations reinstate the complex pathophysiology of endometriosis.

Further, clustering the genes in the knowledgebase, based on their disease associations (excluding endometriosis) (Supplementary Table 1), showed a huge percentage of genes associated with other female reproductive disorders majorly polycystic ovary syndrome (PCOS), reproductive cancers and/or infertility (male and female). Endometriosis is associated with ovarian, endometrioid and clear cell cancer (54–56) and this explains the genetic overlap between the two diseases. Endometriosis-associated genes *GRB14* and *IGF2* play a role in insulin receptor signaling (57). Alterations in the insulin signaling pathway have been observed in endometrial pathologies like endometrial cancers and PCOS (58, 59). In addition to the molecules playing a role in the insulin signaling pathway, the Endometriosis Knowledgebase also consists of several other pleiotropic genes like *IL17A, MUC1*, etc. that have been reported to be associated with PCOS by independent research groups (60–62). Reports on coexistence of endometriosis and PCOS are rare but are present (63). Another interesting observation from the disease-based clustering is an overlap in genes associated with endometriosis and male infertility (azoospermia, oligospermia, etc.). These genes shared between endometriosis and male infertility were associated with secretion of gonadotropins, pubertal development and reproduction.

Coexistence of endometriosis and autoimmune diseases has been reported. Autoantibodies against syntaxin5, tropomyosin3, stomatin-like protein 2, tropomodulin 3, etc. have been identified in patients suffering from endometriosis (4, 64). Several genes associated with endometriosis are also known to share similarities with other autoimmune diseases (65). Gene polymorphisms like *CCL21* (rs2812378) and *HLA-DRB1* (rs660895) polymorphisms have been associated with both endometriosis and RA (66). The matrix metalloproteinases are another class of proteins that are associated with the pathogenesis of both arthritis and endometriosis (67). The ESR2 gene polymorphism was found to be associated with Graves’ disease (68, 69). Independent studies have also indicated a higher prevalence of RA, SLE (70) and Sjogren’s syndrome (71) in patients suffering from endometriosis. The genes present in Endometriosis Knowledgebase and associated with RA, SLE, Graves’ disease and Sjogren’s syndrome (6) were analyzed using Panther. The inflammation-mediated chemokine and cytokine signaling pathway and the apoptosis signaling pathway were the two common enriched pathways in genes associated with endometriosis and the above autoimmune diseases.

Other comorbid conditions associated with endometriosis are endocrine/reproductive (e.g. Premature ovarian failure (POF) etc.), nervous system (e.g. Alzheimer’s, Parkinson’s) and metabolic (e.g. obesity) diseases.

The presence of estrogen, progesterone and androgen receptors in the brain is known (72). Estrogen through the estrogen receptor is known to modify the risk of cancer, neurodegenerative diseases, cardiovascular diseases, insulin resistance, etc. (73). The increased exposure to estrogen leads to endometriosis, and its converse leads to neurodegenerative diseases like Alzheimer’s and Parkinson’s disease. While several studies have suggested a decreased risk of neurodegenerative diseases in endometriosis, a case-control study conducted on Danish subjects indicated a moderately increased risk of Parkinson’s disease in women suffering from endometriosis (74). These conflicting observations need to be investigated further. A genome-wide enrichment analysis between endometriosis and obesity-related trait identified the association of *GRB14* in both these conditions (75).

The diverse pathways and comorbid disease associations present the complex pathogenesis of endometriosis and also necessitate identification of probable disease targets for therapy and effective disease management. A global approach like gene-disease networks that integrates information on genes their associated gene ontologys, pathways and diseases will aid in gaining a better understanding of the pathophysiology of endometriosis. Endometriosis Knowledgebase would be a useful resource for in-depth study on the genetic etiology of endometriosis.

### Predicting novel candidate genes for endometriosis

**Figure 3:**
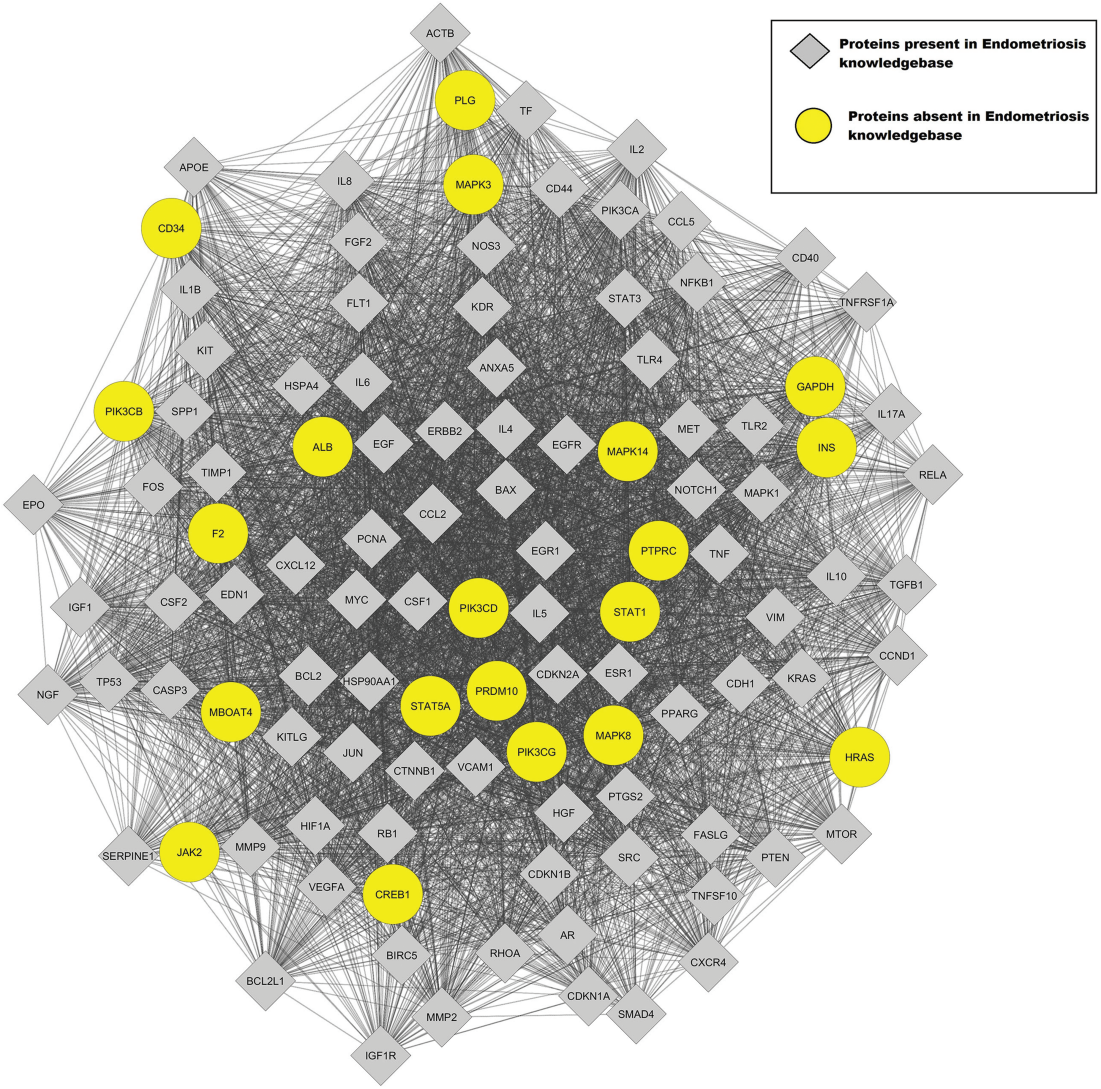
This figure represents the highest-scoring MCODE cluster of the protein–protein interaction network for Endometriosis.

The interaction network for proteins encoded by genes present in the Endometriosis Knowledgebase was imported from the String database using Cytoscape 3.6 (76) with a confidence cut-off of 0.4. The MCODE plugin (77) in Cytoscape was used to find functional modules in the network. The highest-scoring cluster was analyzed for proteins absent in the Endometriosis Knowledgebase but interacting with endometriosis-associated proteins. The genes corresponding to 20 proteins absent in Endometriosis Knowledgebase were identified from cluster1 (Figure 3). Five of these genes, *F2* (78), *INS* (79), *MAPK8* (80), *CREB1* (81) and *STAT5* (82), have been reported to be differentially regulated in patients with endometriosis. *CD34* identified in this study is a marker for endothelial cells that has been reported to be upregulated in endometriosis (83). The *JAK2* protein is part of the JAK2/STAT3 signaling pathway, which is important for migration and invasion abilities of endometriotic cells in endometriosis (84). These genes were included in the Endometriosis Knowledgebase. The Panther Tool was used to delineate the enriched pathways in the remaining 13 genes. The most enriched pathways were the angiogenesis pathway and the Ras pathway that are important for cell proliferation, differentiation and survival. Both these pathways are important for the manifestation of the endometriosis phenotype. The probable role of these genes in endometriosis needs to be determined experimentally.

## Conclusion

Endometriosis Knowledgebase is a useful resource for genes associated with endometriosis. The knowledgebase currently holds information on 831 genes and 1383 associated diseases. These genes associated with endometriosis have been manually curated and the information on the mutation identified/screened; population size, ethnicity and other pathophenotypes observed in the patients have been included in the database. The database also includes information on gene ontology, KEGG pathways and other associated disorders that could be exploited to build gene-disease networks to identify key causal genes or probable new gene targets for endometriosis. Tools to identify homologs of the genes, identify conserved protein domains and motifs and for SNP analysis have been incorporated in the database. Additionally, the analysis section in the database also allows users to input a user-selected gene list from the database to cluster genes based on common diseases, pathways, gene ontology and protein function. A link has also been provided to PANTHER gene list analysis. Network analysis of genes associated with endometriosis was employed and 13 new candidate genes with a probable role in endometriosis were identified. This resource would be useful to both clinicians and researchers working towards understanding the complex genetic etiology of endometriosis.

## Supplementary Material

supplementary_table1_baz062

## Notes

**Database URL:** http://www.ek.bicnirrh.res.in
