# Supplementary material for: Endometriosis Knowledgebase: a gene-based resource on endometriosis
Source: Database (Oxford). 2019 Jun 5;2019:baz062. doi: 10.1093/database/baz062 (PMC6551373; doi:10.1093/database/baz062)
Supplement: supplementary_table1_baz062 [file supplementary_table1_baz062.docx]

| Diseases | Number of genes |
| --- | --- |
| Reproductive Diseases (excluding Endometriosis ) | 442 |
| Cancer | 415 |
| Endocrine Diseases | 369 |
| Nervous System Diseases | 329 |
| Metabolic Diseases | 289 |
| Psychiatric Diseases | 267 |
| Cardiovascular Diseases | 255 |
| Immune Diseases | 250 |
| Developmental Diseases | 199 |
| Musculoskeletal Diseases | 189 |
| Skin Diseases | 182 |
| Respiratory Diseases | 178 |
| Digestive Diseases | 161 |
| Eye Diseases | 143 |
| Urogenital Diseases | 120 |
| Hemic and Lymphatic Diseases | 109 |
| Stomatognathic Diseases | 107 |
| Otorhinolaryngologic Diseases | 41 |

Supplementary Table1: Gene overlap between Endometriosis and other diseases
